# A System Using Artificial Intelligence to Detect and Scare Bird Flocks in the Protection of Ripening Fruit

**DOI:** 10.3390/s21124244

**Published:** 2021-06-21

**Authors:** Petr Marcoň, Jiří Janoušek, Josef Pokorný, Josef Novotný, Eliška Vlachová Hutová, Anna Širůčková, Martin Čáp, Jana Lázničková, Radim Kadlec, Petr Raichl, Přemysl Dohnal, Miloslav Steinbauer, Eva Gescheidtová

**Affiliations:** Faculty of Electrical Engineering and Communication, Brno University of Technology, 61600 Brno, Czech Republic; xjanou09@vutbr.cz (J.J.); xpokor61@vutbr.cz (J.P.); xnovot0v@vutbr.cz (J.N.); xhutov00@vutbr.cz (E.V.H.); xsiruc02@vutbr.cz (A.Š.); capm@vutbr.cz (M.Č.); xlazni09@vutbr.cz (J.L.); kadlec@vutbr.cz (R.K.); xraich02@vut.cz (P.R.); dohnalp@vutbr.cz (P.D.); steinbau@feec.vutbr.cz (M.S.); gescha@feec.vutbr.cz (E.G.)

**Keywords:** bird detection, convolutional neural network, deterrent system, flocks of birds, fruit, fruit crops, starlings

## Abstract

Flocks of birds may cause major damage to fruit crops in the ripening phase. This problem is addressed by various methods for bird scaring; in many cases, however, the birds become accustomed to the distraction, and the applied scaring procedure loses its purpose. To help eliminate the difficulty, we present a system to detect flocks and to trigger an actuator that will scare the objects only when a flock passes through the monitored space. The actual detection is performed with artificial intelligence utilizing a convolutional neural network. Before teaching the network, we employed videocameras and a differential algorithm to detect all items moving in the vineyard. Such objects revealed in the images were labeled and then used in training, testing, and validating the network. The assessment of the detection algorithm required evaluating the parameters precision, recall, and F1 score. In terms of function, the algorithm is implemented in a module consisting of a microcomputer and a connected videocamera. When a flock is detected, the microcontroller will generate a signal to be wirelessly transmitted to the module, whose task is to trigger the scaring actuator.

## 1. Introduction

The protection of fruit crops from raiding flocks of birds constitutes a major problem for fruit farmers and winegrowers. One of the most prominent pests in this respect is the European starling (Sturnus vulgaris), whose immense flocks feeding on fruit in large orchards and vineyards are perfectly capable of ruining the entire harvest [1]. Diverse measures are being taken to prevent this beautiful passerine from attacking fruit fields and wine-growing areas, with the relevant efforts and applicable techniques usually conceived or designed to be physically harmless to the bird. The scaring methods involve mechanical, optical, and acoustic approaches, in addition to utilizing natural enemies.

### 1.1. Mechanical Techniques

The set of mechanical instruments, options, and procedures includes, first of all, nets stretched over the entire orchard or vineyard or wrapped around the individual plants. The main disadvantages of this option rest in the comparatively high purchase cost, rather short service life, and unfavorable ecological impact; moreover, the installation is very complicated, and the labor time to install and remove the nets amounts to approximately 60 h ha^−1^.

Another type of scaring within this group relies on various scarecrows and kites. The former embody a centuries-old, differently configurable tool and can be combined in various ways with the latter, which imitate birds of prey such as the eagle, falcon, and sparrowhawk.

The natural enemies of starlings, blackbirds, and magpies are, for example, larger and more aggressive birds, including eagles, owls, eagle owls, buzzards, and falcons; respected and feared by the pests, these animals have been traditionally used as models for realistic kites. In terms of the actual shape and placement, such models have proved most effective when delivered in 3D and mounted visibly in the vicinity of the tree(s).

Yet another–interesting and modern–alternative consists in using unmanned aerial vehicles (UAVs, drones) to deter a flock that has appeared above the monitored area, whose size must be selected appropriately to the parameters of the drone [2,3]. This category comprises also robotic birds, or robirds [4]; their applicability, however, is markedly limited by the maximum flight time, relevant legislation, particular control and battery charging requirements, and other prominent factors.

### 1.2. Acoustic Methods

In sizeable orchards and vineyards, a hunter’s services are usually of great benefit. The hunter shoots blank cartridges to scare the flock away, the aim being not to kill the birds. The central disadvantage is that the armed guard can watch over only a part of the total area and has to stay active and alert all day, resulting in major financial costs.

Another option consists in utilizing sonic bird repellers, which emit emulated or authentic sounds of raptors or frightened starlings signaling their mates to avoid a dangerous location.

A closely related procedure exploits sounds at frequencies audible and unpleasant to the birds [5]. This principle finds use in, for instance, narrowly directional ultrasonic repellers. Such devices, although often very effective, nevertheless provide optimum performance especially in small, fully or partially enclosed spaces.

By extension, it is also possible to employ gas cannons, such as the Zon Mark 4 Propane Bird Scare Cannon, albeit only at the expense of seriously disturbing persons and animals that live nearby: The guns shoot continuously during the ripening period, creating or increasing the noise burden in the surroundings. The method is comparatively cheap but may gradually become ineffective.

### 1.3. Optical Modes and Instruments

The less costly optical solutions are based on distributing glittering items in gardens or larger growing areas and on external surfaces of houses. This approach deters all types of birds, as the shiny objects disturb them, and the smaller birds are directly scared by the reflections, which resemble raptors’ eyes. Starlings, however, range among intelligent birds and, after a time, may notice that the reflections always occur at the same places and that there is no danger. Another popular tool is the holographic strip [6], a special glossy foil emulating a raptor’s eye; at locations protected in this manner, starlings usually behave very cautiously.

Considering novel trends and progressive technologies, especially in view of their practical applicability and performance, we can emphasize the agrilaser system [7]. In this concept, laser generators emit a beam which is then perceived by the pests as a physical risk or obstacle and makes the birds take off or change their flight paths.

### 1.4. Natural Enemies

In the case of large-scale growers, a viable option is to hire a falconer. As raptors commonly hunt small birds for food, this natural procedure (if regular) embodies a very effective means to defend an orchard or a vineyard against starlings; however, disadvantageously, the method is very time-intensive and costly.

### 1.5. Aims and Objectives

Most of the above-presented methods are problematic in that the pests gradually become accustomed to the disturbance. The issue is especially irritating in the acoustic devices, where, despite the diminished scaring effect, the human and animal exposure to the repelling noises remains at the same levels of intensity. With this difficulty in mind, we designed a system that executes the scaring task only after optically detecting a flock; the triggering signal is communicated to the actuator wirelessly. In the given context, our paper characterizes the hardware and software components of a novel setup that utilizes videocameras and artificial intelligence (AI) to detect flocks of starlings. The entire concept incorporates a scaring element (such as a loudspeaker, a gas cannon, or a laser beam generator) to be activated only when a flock has been detected; thus, the process is not continuous, eliminating undesired sonic disturbance, and this factor constitutes the greatest advantage of the system against regularly marketed options. In this manner, the actual scaring becomes more effective and environmentally friendly thanks to the irregularity of the actuating impulses.

Beyond the Introduction, the paper is organized as follows: Section 2 outlines the state of the art in the field, comparing relevant studies; Section 3 describes the hardware of the system; Section 4 analyzes the applied scaring methodology; Section 5 presents the experiments and their results; Section 6 discusses the general outcomes of the research; and Section 7 embodies the conclusion.

## 2. Previous Research

AI algorithms are currently employed in diverse branches of science and industry, including but not limited to civil [8] and electrical [9] engineering, crude oil drilling or mining [10], and manufacturing control [11].

In software terms, our concept of bird flock detection exploits AI algorithms, utilizing the approaches outlined in previously published studies that focus on AI in the detection of animals. The set of relevant sources comprises, for example, articles [12,13,14,15,16,17], which discuss the monitoring, detection, and classification of birds to prevent their interaction with wind turbines. Most of the monitoring projects described in these studies concern birds in the air. A different technique is proposed by the authors of reference [18]. This study eventually led to the designing of deep learning-based object-detection models using aerial images collected by an unmanned aerial vehicle (UAV). In the bird detection, the authors employ diverse models, including the Faster Region-Based Convolutional Neural Network (R-CNN), Region-Based Fully Convolutional Network (R-FCN), Single Shot MultiBox Detector (SSD), Retinanet, and You Only Look Once (YOLO). Such a model-supported procedure is also applied in source [19]. Another variant of flying bird detection (FBD), formulated for the purposes of aviation safety, namely, to forestall bird - aircraft collisions, is characterized in papers [20,21]; the research utilizes a simplified bird skeleton descriptor combined with an SVM (Support Vector Machine). A similar method appears in article [22], which proposes a feature-based bird detector. By combining the Histogram of Oriented Gradients (HOG) and the Center-Symmetric Local Binary Pattern (CS-LBP) into a feature set, the authors successfully detected crows under various lighting conditions. A dataset of crows, containing a wide range of movement phases, positions, and backgrounds, was prepared and learned with a linear Support Vector Machine (SVM). Further, article [23] introduces a bird detection and classification system exploiting Gaussian and Gabor filters, the HOG, and convolutional neural networks (CNNs). Such networks are employed also by other researchers in ornithology [24], agriculture [25], and air traffic safety centered on collision avoidance [26,27].

Studies [28,29] discuss the behavior of birds in the sky and on water, in addition to evaluating different types of classifiers.

The literature, by extension, includes comprehensive articles addressing both the detection and the scaring of birds [30]. One of these sources is referenced in study [31], which characterizes the prototype of a device that comprises PIR sensors as the detector to track moving birds and uses LC oscillator type colpitts with a piezo ultrasonic sensor as the repeller. An ultrasonic repeller is described also in source [32], whose authors nevertheless follow a markedly more sophisticated path, exploiting machine learning algorithms. The use of an ultrasonic sensor as the actuator in combination with a Haar cascade classifier-based detector is then outlined in article [33].

The method defined in our study, compared to those exposed in the above-mentioned research articles, generally does not rely on detecting individual birds but rather on optimizing learning algorithms to facilitate flock detection. Another significant difference rests in that the design presented herein can involve virtually any actuator, switchable by the wireless module integrated in the system. These aspects together form the actual novelty of the designed setup, as further indicated in Section 1.5 above and the Discussion section below.

## 3. Hardware Components and Functions

**Figure 1:**
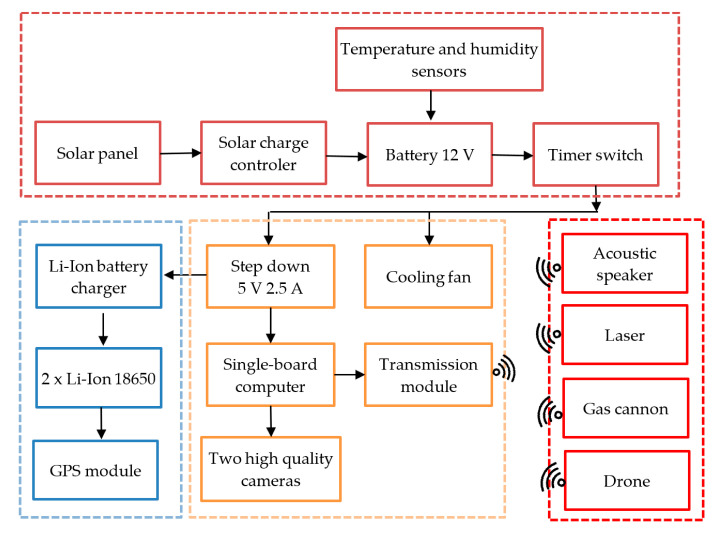
A block diagram of the sample setup for the detection of flying objects.

To scan the monitored space, evaluate the movement, and transfer the detection-related data, we designed an optical detector of moving objects (Figure 1). This detector utilizes a camera system and algorithms to track items in a predefined area, allowing the detection of not only birds and insects but also, if differential image processing is applied, fast processes or effects such as lightnings. The basic element of the detector consists in an NVIDIA Jetson Nano single board-computer, to which two videocameras are connected: One a Raspberry Pi HQ equipped with a Sony IMX477R sensor and a 16 mm f/1.4 PT3611614M10MP lens, and the other an Arducam 8 Mpx USB webcam CCTV 5–50 mm. The combination of two videocameras is used intentionally to deliver fast processing and convenient properties of the images.

**Figure 2:**
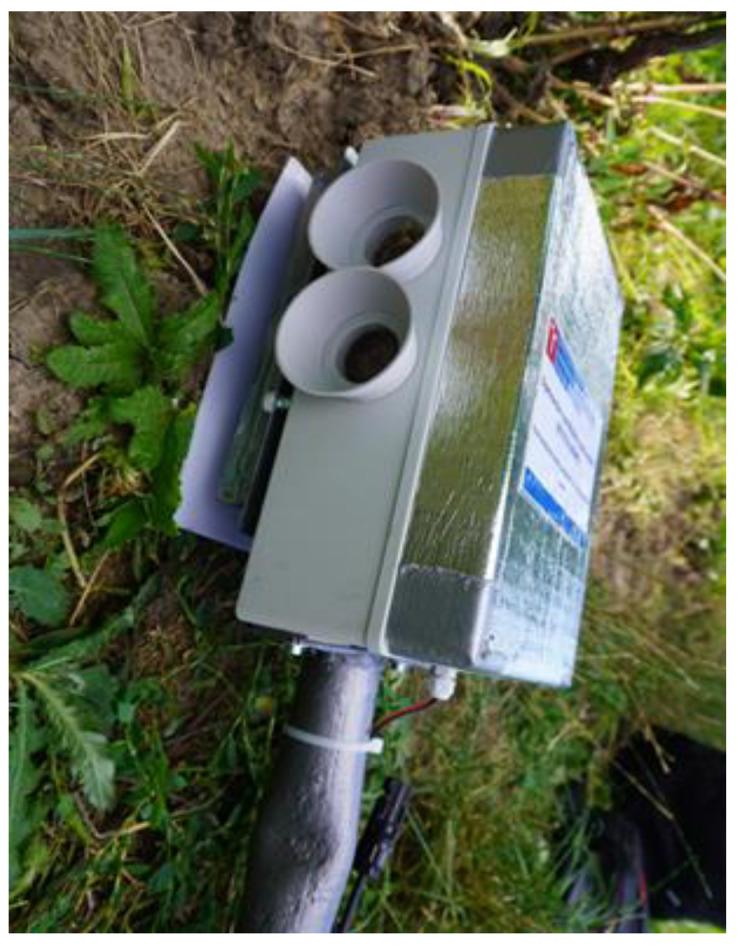
The optical detection and early warning unit to track a flock of birds.

The number of detectors required for a 360° coverage is specified in Table 1. Both of the above-defined videocameras are preset such that a flock can be captured at a distance of 300 m or less; in the Sony IMX477R, the minimum zoom value has to be selected in advance. In addition to the distance, the parameters that define the coverable area include the horizontal field of view (FOV). Before the actual monitoring, the devices are inserted in 3D-printed visors (Figure 2).

Considering the fact that the optical detector was to be tested in a vineyard with no connection to the electricity grid, we had to assemble a small island network [34]. For this purpose, we employed a photovoltaic panel exhibiting the following parameters: Max power 100 W, open circuit voltage (Voc) 21.6 V, short circuit current (Isc) 6.11 A, max power voltage (Vmp) 18 V, and max power current (Imp) 5.55 A. The electricity obtained from this panel was transferred to the solar charger controller and then fed directly to the devices or stored in the battery. In In island systems, a key factor consists in low consumption; we therefore implemented in the design a time delay switch to activate the entire setup an hour before sunrise and to shut it off an hour after sunset. Thus, we enabled the installed optical detector to operate over the complete grape harvest period in the Czech Republic.

The electricity to the microcomputer is supplied by a 5 V voltage changer having the maximum current of 2.5 A; the videocameras receive power from the microcomputer. Separate feeding is secured for the module measuring the temperature and humidity, which, thanks to the attached SIM card, transmits the acquired data over an LTE network every 30 min. The reason for this configuration is that we need to know the temperature and humidity inside the sealed box, intending to evaluate the data before further use. To keep the internal spaces of the optical detector cooled, we fabricated a heat sink and mounted it to the rear wall of the module; the air circulation is guaranteed by a cooling fan running on a voltage of 12 V. As vineyards are generally warm and sunny, and the setup is exposed to intensive sunlight, the surfaces of the plastic box were covered with a special reflective foil.

The two videocameras capture the position of the flock, allowing the actuator to be wirelessly triggered to execute the scaring task at the planned location. The wireless signal is transferred via WiFi, using an ESP8266 module. The actual receiver comprises only the power supply, ESP8266 module, and a relay to switch on the feeding (up to 10 A) and signal lines of the actuator (an ultrasonic repeller or a gas cannon).

**Figure 3:**
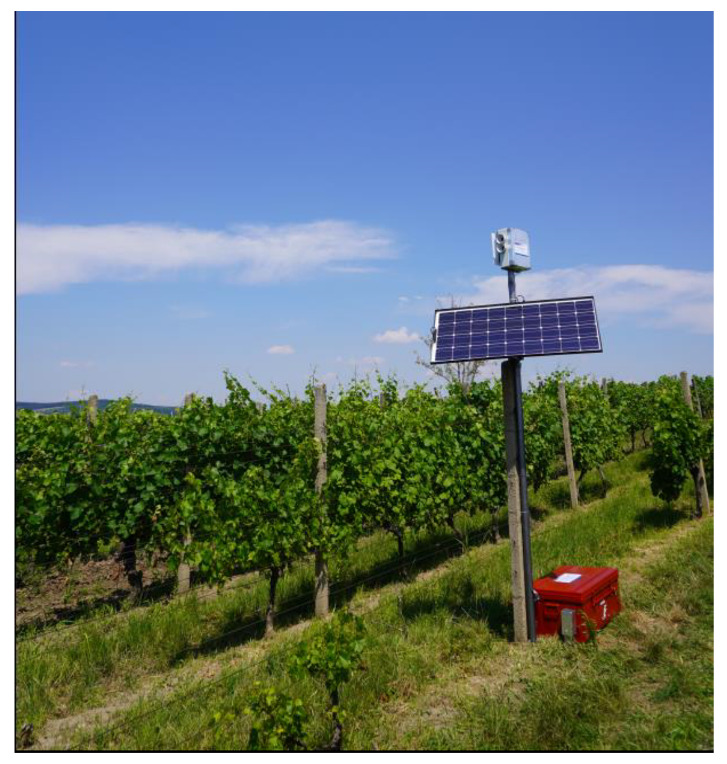
The complete setup located at a vineyard in Bořetice, Moravia, the Czech Republic.

The optical detection and early warning unit is detailed in Figure 2; the complete assembly, erected at a vineyard in the village of Bořetice, Moravia, the Czech Republic, is then visualized in Figure 3.

## 4. Methods for the Detection of Moving Targets

When investigating the possibilities of the visual detection, we employed diverse image processing techniques combined with deep learning. The algorithms were implemented in the module represented in Figure 1, Figure 2 and Figure 3.

At the initial stage, we traced any and all visual activity by utilizing a differential algorithm in the detection of movement [35,36]. The captured set included all items that had changed their positions by at least one pixel between two instances of imaging. The images contained individual birds, bird flocks, insects, and other objects. In total, we executed approximately 300,000 detection shots, and these then allowed us to use in the research a corresponding number of images with detected items. From such a perspective, the differential method for the detection of moving objects appears to be wholly insufficient in monitoring only a single concrete item; in our case, however, the object is a flock of birds. The acquired images nevertheless embodied a good input to support the subsequent application of deep learning. To prepare for this procedure, we classified and annotated the images, eventually obtaining a set of training images. The result was a classification model implemented in a microcomputer to facilitate the visual detection of bird flocks.

### 4.1. Image Capturing

**Figure 4:**
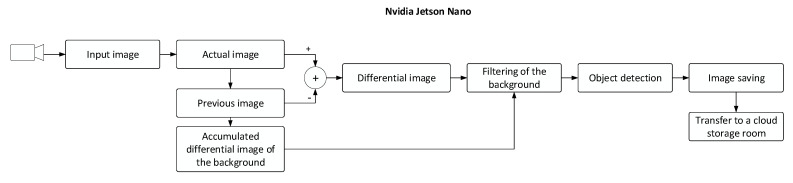
A flow chart characterizing the differential method (the input, actual, previous, and accumulated images); the differential image of the background; the differential image; the filtering of the background; object detection; image storage; transfer to a cloud storage room.

**Figure 5:**
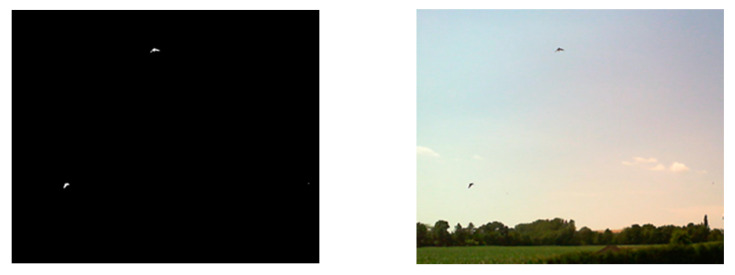
A binary differential image (**left**) and a highlighted region around a detected object (**right**).

This section characterizes the image capturing procedures in greater detail. At the initial stage of the process, we formed a set of images capturing objects moving in the vineyard, utilizing an algorithm based on the differential method complemented with background image filtering (Figure 4). This technique exploits the principle of a movement detector capable of eliminating false detection caused by the quivering of grapevine leaves or the green vegetation in the vicinity. By simply subtracting the current image from the previous one, we yield a differential image, which is then converted to a binary image resulting from a predefined threshold. Simultaneously, an accumulated differential image of the background is being created to be later subtracted from the differential image; thus, we ensure the filtering of the background. Furthermore, object detection via the recognition of binary contours is in progress; during the operation, any image containing a positively detected item is stored and sent to a cloud storage room. A binary differential image and a highlighted region around a detected object are shown in Figure 5.

**Figure 6:**
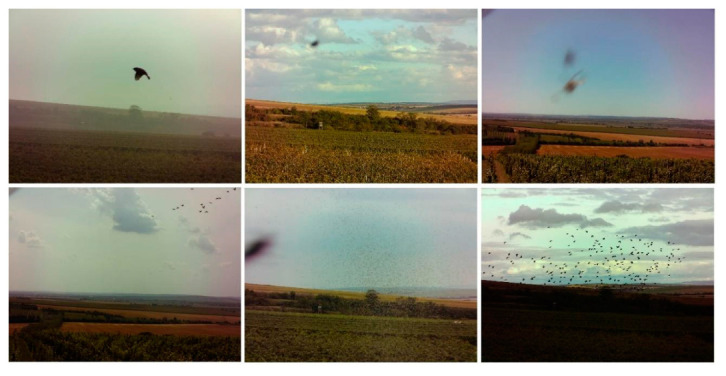
Examples of images capturing the activity in a vineyard.

**Figure 7:**
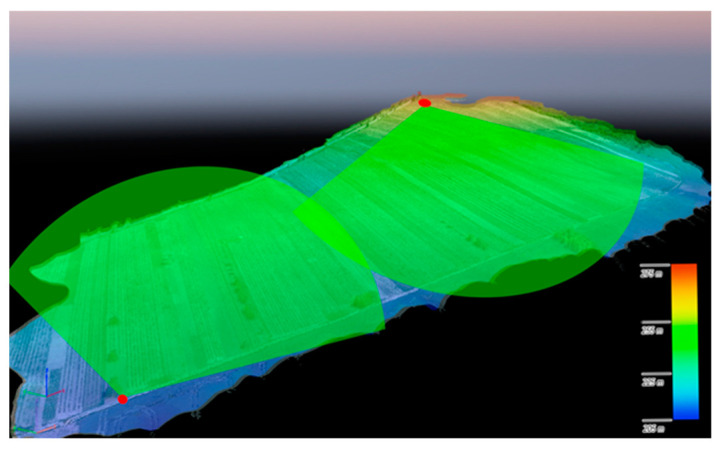
A special content 3D map: the placement of the optical detectors.

The unit visualized in Figure 2 is capable of detecting bird-size objects in real time, up to a distance of approximately 300 m. If movement is recognized, the image is sent to a cloud storage room. The images collected during our experiment comprised multiple false detections due to insects, and this effect can be considered the greatest disadvantage of the applied differential algorithm. The technique, however, enabled us to gather a sufficient number of images (see Figure 6 for examples), which then found use at the next stage, in training the classifier via machine learning methods. The data collection took place at two wineries, one based in Bořetice and the other in Popice (both in Moravia, the Czech Republic). The altitude profile of the vineyard and the distribution of the optical detectors are represented in Figure 7, where each of the red spots represents three detecting modules that cover the areas highlighted in green.

**Figure 8:**
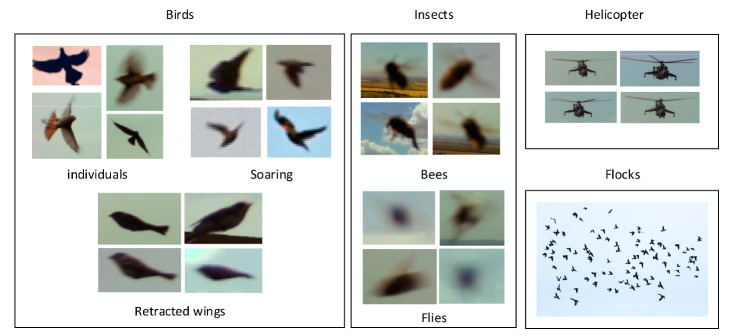
Categorizing the collected images (Individual birds: spread/retracted wings; insects: bees and flies; helicopters; flocks).

The bulk of data collected with the detection algorithm comprised approximately 300,000 images, from which 993 were selected for further processing. Subsequently, the individual items in each image were annotated and categorized, with the birds classified into more specific subsets to demonstrate their distinctive visual in-flight characteristics (Figure 8). During the machine learning phase, the images were assorted again, roughly at the ratio of 8:1:1, into the training, validating, and testing subgroups (see Table 2, in which the individual data match the counts of detected objects). For our purposes, the term *flock* denotes not less than five birds flying together.

### 4.2. Machine Learning Algorithm

**Figure 9:**
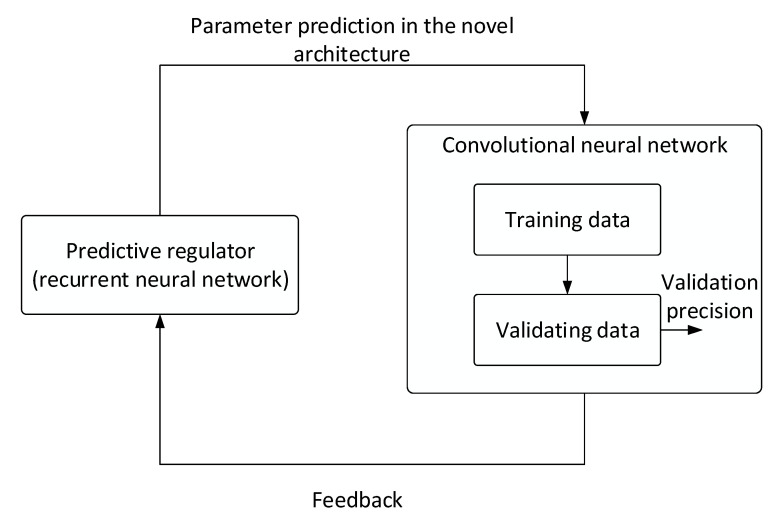
The principle of the machine learning algorithm to assemble a convolutional neural network by utilizing the cloud-based service *Google AutoML Vision*.

To facilitate the recognition of objects in the images, we employed the cloud-based instrument *Google AutoML Vision* [37,38,39,40], which delivers an algorithm for the training of neural networks. The *AutoML* service utilizes recurrent neural networks to search for an optimum neural network architecture (applying a procedure referred to as neural architecture search) [41]. The algorithm (Figure 9) suggests the basic set of hyperparameters as well as the counts of layers and nodes to be required from the convolutional neural network that is being searched for. In the next iterations, the individual parameters are specified more precisely by the feedback. This process then repeats until the algorithm has gradually identified the best-fitting optimum architecture for the concrete dataset. The desired model is then selected from the set of assembled neural networks in such a manner that it possesses optimum properties, including sufficient accuracy and detection sensitivity.

### 4.3. Parameters of the Classifier

**Figure 10:**
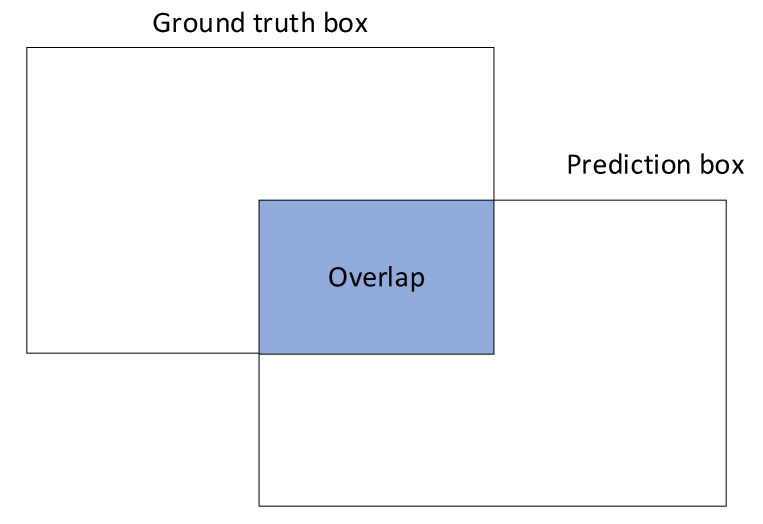
The overlap between the predicted ground truth and a detected object.

The classifier was trained with the cloud-based tool *Google AutoML Vision* featuring separate validation and testing sets. To allow the implementation, we used an exported TensorFlow Lite model after the classifier has been trained. The results delivered by the classifier had followed from a comparison of the detection algorithm with the annotated test images. The prerequisite for evaluating a correctly detected object (true positive) was an overlap of at least 50% with the ground-truth template (Figure 10).

At this point, we would like to define Intersection over Union (IoU), also called the Jaccard index. It is a metric that evaluates the overlap between the ground-truth mask (*GT*) and the predicted mask (*PD*). We can use IoU to determine if a given detection is valid or not.

IoU is calculated as the area of overlap/intersection between *GT* and *PD*, divided by the area of the union between the two, that is:

(1) $$IoU=\frac{Area\;of\;overlap}{Area\;of\;union}=\frac{GT\cap^{\;}PD}{GT\cup^{\;}PD}.$$

Other parameters enabling correct evaluation of the images were as follows:

*Confidence rate*—virtually errorless detection is achievable but only at the expense of low sensitivity, and vice versa. To carry out the testing, we set the confidence rate to 30%.

The detection *precision* rates, presented in Table 3, denote the proportion of true-positive detections within the entire set of detected objects. We thus have the following:

(2) $$Precision=\frac{True\;positive}{True\;positive+False\;positive}.$$

A further criterion consists in the detection sensitivity value (*recall*):

(3) $$Recall=\frac{True\;positive}{True\;positive+False\;negative}.$$

The *F1 score* then evaluates the harmonic average of precision and sensitivity, via:

(4) $$F1=\frac{2\cdot True\;positive}{2\cdot True\;positive+False\;positive+False\;negative}.$$

Finally, we evaluated the *error rates* in all classes of the trained classifier. This quantity is given as the proportion between all misclassified objects and the total number of detected objects in a concrete class, expressed through

(5) $$Error\;rate=\frac{False\;positive+False\;negative}{False\;positive+False\;negative+True\;positive+True\;negative}.$$

## 5. Results

**Figure 11:**
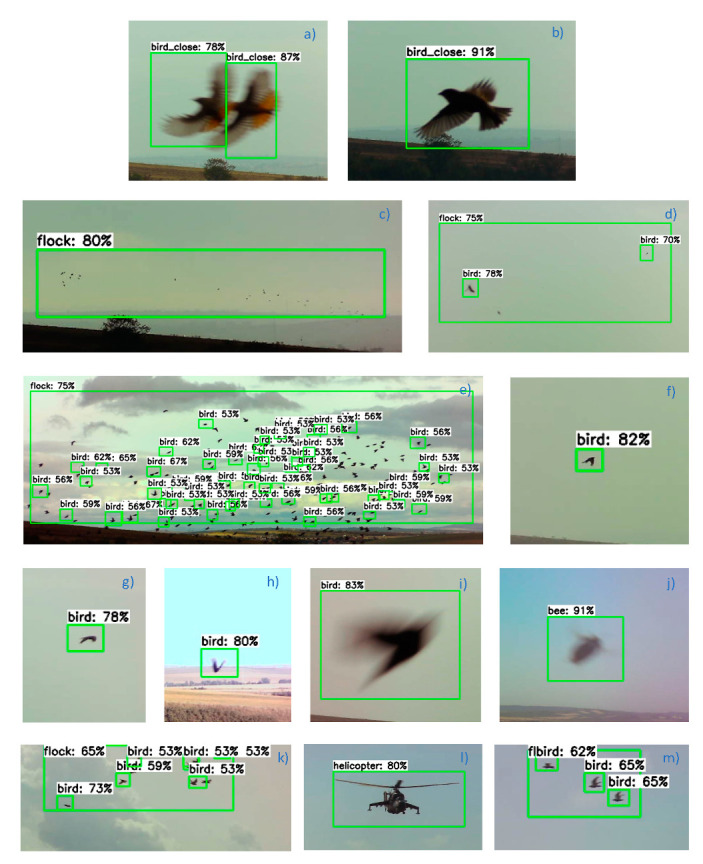
The detection outcomes in individual objects.

The figure below demonstrates the object detection outcomes obtained in testing the designed optical detector. Figure 11a,b,i show some of the birds that were tracked near the movement-sensing videocameras, at a distance of about 10 m. The visualized procedures also include the detection of flocks Figure 11c–e,k at various distances and the tracking of objects correctly defined as insect Figure 11j or a helicopter Figure 11l. The percentage numbers in the captions to the green frames that mark the detected items express the confidence scores; in terms of this parameter, the designed optical detector algorithm is set such that if the optical unit detects a flock at a confidence score of at least 30%, the actuator will be triggered to execute a scaring task.

The correctness and error rates related to the results obtained during the testing of the trained classifier are summarized in Table 3. The Table compares the performances of the detection algorithm with the ground truth (*GT*) set, which represents objects annotated in advance. Correctly evaluated items are introduced within the true positive (*TP*) set. Further, the false negative (*FN*) detections group comprises all items that were not evaluated as belonging to a given class but actually fit within it. The false negatives are caused by the lower sensitivities accompanying a class. Lastly, objects identified as false positive (*FP*) were detected under a class but, in reality, do not fit; such detections generate false alarms and are undesirable in the application of a classifier.

To offer a more comprehensive idea of the behavior of the classifier, we assembled a relevant confusion matrix. This matrix is bound to the false positive (*FP*) detections; such detections, in turn, comprise false-positive misclassifications that match a different class on the one hand, and those that do not fit within any class on the other. As shown in Table 4, confusing a detected object with another class is a rare, nearly nonexistent scenario. In most cases, the detection type involved is *FP*, with the object being outside all of the *GT* subclasses. Thus, we can claim that the classifier is very resistant to being confused between the classes.

Table 5 describes the resulting detection parameters obtained from the individual objects within the testing sets. The numbers are based on the data in Table 3, indicating the detection *precision*, *recall*, *F1 score*, and *error rate*.

## 6. Discussion

To optimize the AI algorithms, we first acquired approximately 300,000 images to choose items suitable for further processing. After this pre-selection, the testing dataset comprised 134 images containing 1008 ground truth objects. Within the presented results, the precision rates obtained in the birds, insects, and flocks amounted to 83.4%, 65.8%, and 100%, respectively. The set of bird flock images captured 35 objects; of these, 33 items were detected correctly, and two exhibited falsely negative classification. At this point, it should be emphasized that the resulting classifier evaluated the flocks correctly up to a distance of 300 m.

As regards the recall parameter, the rates in birds and insects equaled 83.4% and 67.9%, respectively. The lower sensitivity values were due in particular to the lower sensitivity (confidence rate) of the classifier; this effect arose from the relatively small dimensions of the objects. In bird flocks, however, the sensitivity reached 94.3%, mainly because such formations are better definable with ground truth, to become detectable more effectively (Figure 10). The *F1 score* indicator exhibited the value of 97.1% when applied to bird flocks; the capturing of potentially dangerous flocks can thus be considered successful.

In bird flocks, the *error rate* remained at 5.7%; the other classes, however, produced higher rates, owing to the greater uncertainties of the classifier. This evaluation was influenced by a larger number of false negatives, which were caused mainly by the greater distance of the object from the camera, resulting in a poorer detection performance in smaller birds and insects.

The most significant obstacles to correct classification rested in the overly small dimensions of the objects, caused by a long distance from the videocamera, and inferior light conditions. Conversely, large flocks or even small formations of several individuals are detectable with very good results. The reduced precision and sensitivity of detection then arise especially from the remote position of the objects being detected. In practical terms, it should be pointed out that the test involved randomly selected images. In reality, a flock is captured in multiple images to be evaluated; such a process then ensures sufficient sensitivity.

The number and placement of the cameras depend on not only the parameters of the applied videocameras but also the area and altitude profile of the vineyard being observed. Typically, vineyards in the region consist of long narrow stripes of land that are diversified according to the owner and/or vine varietal. In the basic device placement scheme, a videocamera becomes the center of a circle having the radius of 300 m. To cover the full range of 360°, we need at least 8 such sensing modules. Alternatively, it is possible to plan triangular or romboidal detection segments.

Most of the papers referred to in Section 2 discuss detecting individual birds; in this procedure, the proportion of the bird’s size to its distance from the videocamera is vital. The algorithms proposed herein, however, were designed to deliver detect flocks rather than individuals, and provide high operational sensitivity for the given purpose. The sensitivity rate could nevertheless be increased even further by reducing the distance between the optical detector and the actual flock. Bird flock detection is discussed in the relevant paper [2], where other algorithms are employed together with the UAS-based process; thus, the detection parameters are then somewhat worse than those delivered through our research.

Generally, neither the commercial manufacturers nor the literature have utilized the above-described approach to date in dedicated scaring of pest bird flocks (as opposed to individual birds), whose raids on ripening fruit significantly reduce the planned harvest.

The design that best resembles ours is the AVIX, a bird detection setup exploiting AI. According to previous articles, this system is capable of distinguishing between birds, humans, and other objects. In functional terms, the birds are scared away by a green laser beam. As regards our design, it is appropriate to stress again at this point the fact that the actuator will not be triggered when the system has detected an individual bird but only after an entire flock has been identified. The innovative concept, moreover, allows the user to incorporate in the system multiple optional scaring components, including acoustic equipment (loudspeakers or gas cannons), lasers, and drones.

Interestingly, a comparison between the design proposed herein and commercial repellers, such as gas cannons and acoustic devices, will show that our approach eliminates a major portion of the disturbances generated by standardly marketed instruments, thus bringing substantial benefit to both humans and animals living in the vicinity of the monitored area. Such a capability stems from the adopted technology, where–unlike the other, presently available options–the actuating impulse is sent exclusively when a flock has been found. The main asset of the novel design rests in that the birds cannot become accustomed to the scaring process, by contrast to the continuously repeated effects [3] triggered regardless of whether a flock is within reach or not. 

The limitations to our approach encompass, above all, the reduced FOV of the cameras; thus, the overall number of applied detecting and scaring modules has to be suitably increased in the future.

## 7. Conclusions

We designed and characterized an AI-based system to allow timely detection of bird flocks that damage ripened fruit. To approach errorless detection, i.e., to prevent false negativity, we set the confidence rate to 30%. At higher rates, the algorithm in the microcomputer will wirelessly activate the actuator, which then will initiate the bird scaring procedure. As regards the recall value (sensitivity), the level achieved in the procedure is 94.3% and rises further when the flock moves towards the camera. At flock distances exceeding approximately 300 m, falsely negative results begin to appear. In terms of the structure of the actual system, the paper discussed both the software and the hardware, as they together ensure two significant benefits. The first of these advantages consists in that the scaring will materialize only when a flock arrives, meaning that no persons or animals in the area are disturbed by continuous sounds emitted by conventional gas guns or acoustic devices. The newly proposed technology also reduced the adaptability of a bird flock to the scaring sounds, an effect that occurs in commercial acoustic devices. Another benefit then rests in reducing the electricity costs, thanks to the scaring modules being switched only after a flock has appeared. Thus, the battery charging requirements in the scaring device are lessened; in the photovoltaic process, a smaller area for the panel to ensure sufficient charging in necessary.

## Figures and Tables

**Table 1:** The technical specifications of the applied cameras.

Camera	Sony IMX477R + PT3611614M10MP		Arducam 8 MP IMX179AF		
Zoom	Horizontal FOV	Number for 360° coverage	Zoom	Horizontal FOV	Number for 360° coverage
Min	44.6°	8.1	Fixed	64.5°	5.6
Max	21.8°	16.5			

**Table 2:** The categories and their counts in the training, validation, and testing subgroups.

Category		Training	Validation	Testing
Insects	Flies	442	41	43
	Bees	36	7	4
Birds	Individuals	2308	445	228
	Retracted wings	55	6	6
	Soaring	160	5	3
Flocks		79	29	26
Helicopters		11	2	4

**Table 3:** The assignment of the various types of objects to the *GT*, *TP*, *FP*, and *FN* sets.

	Ground Truth (*GT*)	True Positive( *TP*)	False Negative (*FN*)	False Positive (*FP*)
bird	898	749	149	145
bug	71	48	23	24
flock	35	33	2	0
helicopter	4	4	0	0

**Table 4:** The confusion matrix for multi-class classification.

	Predicted Class				
**Ground truth**		Bird	Bug	Flock	Helicopter
	Bird	749	1	0	0
	Bug	3	48	0	0
	Flock	1	0	33	0
	Helicopter	0	0	0	4

**Table 5:** Comparing the *precision*, *recall*, *F1 score,* and *error rate* between the individual classes.

	*Precision*	*Recall*	*F1-Score*	*Error Rate*
Bird	83.4%	83.4%	83.4%	28.2%
Bug	65.8%	67.6%	66.7%	49.5%
Flock	100.0%	94.3%	97.1%	5.7%
Helicopter	100.0%	100.0%	100.0%	0.0%

## Data Availability

Not applicable.
